# Assessing the quantities of legume-based products: A dataset of conversion coefficients into a comparable dry-legume equivalent unit

**DOI:** 10.1016/j.dib.2024.111222

**Published:** 2024-12-10

**Authors:** Marine Spiteri, Lola Pedrini, Agathe Thierry, Valérie Orozco, Olivier de Mouzon, Zohra Bouamra-Mechemache

**Affiliations:** Toulouse School of Economics, INRAE, University of Toulouse Capitole, 1 Esplanade de l'Université, F31000 Toulouse, France

**Keywords:** Legumes, Proportion, Consumption, Food products, Dry, Processed, Recipe, Technical coefficient

## Abstract

Measuring the consumption of processed foods made from a common raw agricultural ingredient requires to make quantities comparable, by converting them in raw product equivalent. This conversion also allows to compute total quantities. In the case of legumes, the challenge is to take into account a wide diversity of final products including packaged dry legumes, processed legumes and products cooked from legumes and other ingredients. While the total quantity of the final product purchased or consumed is easily available, the corresponding quantity of dry legumes used to make the final product is not.

We create a dataset of technical coefficients to convert quantities of final legume-based products into dry-legume equivalent unit. For this purpose, we first list all legume-based products purchased in French retail stores from 2002 to 2019. Those products were identified in our primary data source, the Kantar Worldpanel data. Then, for each final legume-based product, we rely on information from existing food databases, literature and products labels to build a coefficient based on three intermediary technical sub-coefficients. The first sub-coefficient measures the proportion of cooked legumes used in each product. The second sub-coefficient adjusts the quantities for products that lose water during processing (such as dehydrated or baked products). Finally, the third sub-coefficient is a conversion coefficient, used to express the quantities of legumes purchased in dry-legume equivalent.

This dataset centralizes technical conversion coefficients that are useful to analyze global French legume markets, regardless of the type of product purchased (dried legumes, canned legumes, meals...). These coefficients also allow to infer quantities at the upstream agricultural production level.

Specifications Table

**Table:** 

Subject	Food science
Specific subject area	QuantiLeg dataset provides technical coefficients to convert quantities of final legume-based products into dry-legume equivalent unit
Type of data	Table (csv file) Raw, analyzed
Data collection	The list of legume-based food products sold in France is extracted from the Kantar Worldpanel 2002-2019 database. Legume-based products in all their diversity, from packaged dry legumes to processed products, are grouped according to their characteristics in order to assign them a single recipe. Based on the literature, food databases and product label information, we have established a technical conversion coefficient, based on three sub-coefficients: “Proportion of legumes used in the final product”, “Rehydration coefficient” and “Dry-legume conversion factor”.
Data source location	Toulouse School of Economics, INRAE, University of Toulouse Capitole, Toulouse, France 1 Esplanade de l'Université 31080 Toulouse Cedex 06, France Primary data sources: Food products included are from the Kantar Worldpanel database covering purchases in France between 2002 and 2019 – *Retrieved from Odalim INRAE* (*Secondary source, project N°75*): “*TNS, Worldpanel 2002.V1 - 2004*” “*TNS, Worldpanel 2003.V2 - 2005*” “*TNS, Worldpanel 2004.V2 - 2007*” “*TNS, Worldpanel 2005.V2 - 2008*” “*TNS, Worldpanel 2006.V3 - 2008*” “*TNS, Worldpanel 2007.V3 - 2011*” “*TNS, Worldpanel 2008.V2 - 2011*” “*TNS, Worldpanel 2009.V1 - 2011*” “*Kantar, Worldpanel 2010.V1 - 2012*” “*Kantar, Worldpanel 2011.V4 - 2013*” “*Kantar, Worldpanel 2012.V2 - 2014*” “*Kantar, Worldpanel 2013.V3 - 2016*” “*Kantar, Worldpanel 2014.V1 - 2016*” “*Kantar, Worldpanel 2015.V1 - 2017*” “*Kantar, Worldpanel 2016.V1 - 2018*” “*Kantar, Worldpanel 2017.V1 - 2019*” “*Kantar, Worldpanel 2018.V1 - 2020*” “*Kantar, Worldpanel 2019.V1 - 2021*” Where “i” of “Worldpanel YYYY.Vi - ZZZZ” is the version number of the data, YYYY is the year of data collection and ZZZZ is the year the data was received by Odalim INRAE.
	Food data: • Recipe database from INCA2 survey (2008). File “*Recettes inca2 V1 - recettes inca2 a plat V1.1 - 16.csv*”. Retrieved from Odalim INRAE (*Secondary source, project N°75*) • Oqali database (https://www.oqali.fr/donnees-publiques/base-de-donnees-oqali/, last consultation November 2023) • Open database Open Food Facts (https://fr.openfoodfacts.org, last consultation November 2023) • Manufacturer and retailer websites (last consultation November 2023) • Open dataset ClasSFood (https://doi.org/10.57745/TRL1XH)
Data accessibility	Data are accessible in a public repository. Repository name: Recherche Data Gouv (https://recherche.data.gouv.fr/fr) Data identification number: doi:10.57745/QZDIUT Direct URL to data: https://doi.org/10.57745/QZDIUT
Related research article	*None*

## 1 Value of the Data

• Our data are useful for measuring the consumption of legumes and its evolution in the context of a sustainable transition to plant-based diet.
• Our data make it possible to compare and aggregate consumption volumes of legume-based products, whether they are sold dried, canned or used as ingredients in cooked food products.
• Our work enables to show the evolution pattern of legume-based products consumption over time in France.
• This type of data is useful for all scientists that work on food consumption (data purchases or surveys on consumption habits), for both macro analysis (French market) and micro-ones (household or individual consumption). Working with dry equivalent quantities also allows to enrich research considering agricultural production data.
• The quality of the data is guaranteed by the application of an ordered and precise protocol described below.

## 2 Background

The role of legumes is crucial in the transition towards sustainable diet. As part of the Brightspace project^1^, we aim to analyze the potential development of legume production through an increase in demand. To this end, we measure the evolution of French legume markets over a long period of time.

Our study covers all legume-based food products purchased in French retail stores. Legume-based products are very diverse: from dry to processed or cooked as an ingredient. The QuantiLeg dataset is needed to convert the quantity of final products purchased into dry-legume equivalents in order to compare quantities by species and to analyze trends. The evolution patterns of legume consumption are computed from the derived quantities.

## 3 Data Description

The dataset [1] consists of one csv file denoted as “QuantiLeg_YYYYMMDD_vXX.csv”, where YYYYMMDD stands for the file generation date and vXX stands for the version number. It lists 62 aggregates of legume-based products (canned legumes, dried legumes, cassoulet…) that consumers can face in stores. Note that each aggregate includes several products with a similar recipe especially concerning the legume content, even if some heterogeneity can exist (e.g. different brands or sub-products). Each legume-based product aggregate is defined by a unique identifier “*Id_Product*” and described with a French name (column “*Product_fr*”) and its English translation (column “*Product_eng*”).

The dataset includes, for each aggregate, as many rows as there are distinct legumes in the product. For example, the dataset provides two rows for a salad made with lentils and tofu: one for the lentils, another one for the tofu. The variable “*Id_pulse*” is an identifier of the legume used, so that each combination of variables “*Id_Product*” and “*Id_pulse*” is unique. The associated variable “*Processing_Type_fr*” (and its English translation “*Processing_Type_eng*”) describes the species of legume used (bean, chickpea…) and its type of processing in the product (dried, soaked, steam cooked…).

We also assign to each legume-based product aggregate a category of the ClasSFood^2^ nomenclature [2] at two different levels of detail: an aggregated level (“*Agg_Classfood_Cat_fr*”), translated into English (“*Agg_Classfood_Cat_eng*”), and its ClasSFood identification number (“*Id_Agg_Classfood_Cat*”); and a more detailed level (“*Classfood_Cat_fr*”), translated into English (“*Classfood_Cat_eng*”), and its ClasSFood identification number (“*Id_Classfood_Cat*”).

The other columns provide the coefficients required to convert quantities of final legume-based products into dry-legume equivalent unit:

• the sub-coefficient of legumes proportion in the product (“*Legume_Share*”)
• the source of information we used to construct the sub-coefficient “*Legume_Share*” (“*Source_Legume_Share*”)
• the sub-coefficient to adjust the quantities for products that lose water during processing (“*Rehydration_Coeff*”)
• the sub-coefficient to convert the quantities of legumes in dry-legume equivalent quantities (depending on the type of processing of the legumes) (“*Dry_Conversion_Coeff*”)
• a global coefficient which is the product of the first three technical sub-coefficients (“*Total_Coeff*”)

An overview of the table file is given in Table 1.

**Table 1:** Structure of the dataset.

Column Name	Type of variable/ Possible values	Short description
Id_Product	Integer (1, …, 62)	Identification number of the legume-based product aggregate
Product_fr	Text	Name of the legume-based product aggregate (French)
Product_eng	Text	Name of the legume-based product aggregate (English)
Id_pulse	Integer (1, 2)	Identification number of the legume used in the product aggregate
Processing_Type_fr	Text	Species of legume used (bean, chickpea…) and its type of processing in the product (dried, soaked, steam cooked…) (French)
Processing_Type_eng	Text	Species of legume used (bean, chickpea…) and its type of processing in the product (dried, soaked, steam cooked…) (English)
Id_Agg_Classfood_Cat	Integer	Identification number of the ClasSFood category at the aggregated level
Agg_Classfood_Cat_fr	Text	Name of the ClasSFood category at the aggregated level (French)
Agg_Classfood_Cat_eng	Text	Name of the ClasSFood category at the aggregated level (English)
Id_Classfood_Cat	Integer	Identification number of the ClasSFood category at the detailed level
Classfood_Cat_fr	Text	Name of the ClasSFood category at the detailed level (French)
Classfood_Cat_eng	Text	Name of the ClasSFood category at the detailed level (English)
Source_Legume_Share	Text - INCA2 – food ID - Open Food Facts - Oqali - Manufacturer/Retailer website - Based on assumptions - Inferred from literature	Source of information for the sub-coefficient “*Legume_share*” For INCA2 source of information, “food ID” stands for the identification number of the food/generic recipe used in INCA2 recipe data
Legume_Share	Percentage (2.0% to 100.0%)	Sub-coefficient reflecting the proportion of legumes in the product
Rehydration_Coeff	Percentage (100.0% to 1000.0%)	Sub-coefficient to adjust the quantities for products that lose water during processing
Dry_Conversion_Coeff	Percentage (9.3% to 340.0%)	Conversion sub-coefficient to express the quantities of legumes in dry-legume equivalent quantities
Total_Coeff	Percentage (0.8% to 100.0%)	Global coefficient which is the product of *Legume_Share, Rehydration_Coeff* and *Dry_Conversion_Coeff*

## 4 Experimental Design, Materials and Methods

We follow the definition of legumes provided in Didinger and Thompson [3] and we focus on the six main pulses in France (lentils, beans, chickpeas, flageolets, split peas and broad beans) used for food consumption, which in Europe refers to protein crops, and on soy-based products. However, we exclude products containing legumes in the form of soy oil. This is because we are interested in the protein transition of the diet and soy oil does not contain protein. We also exclude soy sauce in meals because the associated quantity of soybean can be neglected.

To identify legume-based products sold in French retail stores, we rely on Kantar Worldpanel (KWP) purchases database. These data are one of the main reliable sources of French household food purchases and their fairly precise and fine scale allows to match them to other databases on a similar or more aggregated scale. Therefore, they have previously been used by many scientists, such as economists and nutritionists, to analyze French household consumption [4]. For this research project, we have access to the KWP database from 2002 to 2019. The panel is composed of households from mainland France and is representative of the whole population. Each year, between 10,000 and 30,000 panellists describe their daily food purchases. The available purchases concern food products that are purchased in France. They can be produced in France or imported from other countries. Moreover, the data only record food products bought for "home consumption", thus excluding "out of home" consumption. Each observation in the data panel corresponds to one act of purchase, and there are between 7 and 19 million observations each year. In addition to the quantities purchased, products are described through a set of detailed descriptive variables including the type of dish, the storage and the brand.

In the KWP database, legume-based products take various types of processing such as dried, canned, frozen, cooked as meals, seasonings, desserts, drinks, or bakery. Identifying them is thus tricky. Therefore, we base our selection of legume-based products on their descriptive variables such as “Type of dish” or “Storage” and on the following assumptions:

H1: All products containing the name of a legume in their description are included in the list of products to be studied (soy, bean, lentil salad…).
H2: All cooked products associated with recipe that traditionally contains legumes, such as cassoulet^3^, are assumed to contain legumes (except specific indications).
H3: Thai, Chinese, Vietnamese or Asian recipes, salads, soups or vegetable mix are supposed to be made with soy sauce and/or bean sprouts. We exclude those products because soy from soy sauce in meals is neglected, and bean sprouts are not pulses.
H4: Without any presence of information on legumes, products described as containing vegetables are not included. However, following Rödl's definition of meat alternatives^4^ [5], an exception is made for products that are potential meat imitates (i.e. we keep “vegetable galette” but not “vegetal mix”).
H5: In the KWP data, some recipes lack the precision to determine whether they contain legumes, and what species of legumes (i.e. “vegetable galette”) they contain, so we use several other databases to obtain this information.

Once this selection has been made, we aim at assessing the quantities of consumed legumes. For this purpose, we need to transform the purchased quantities recorded in KWP data so that they can be comparable. This requires detailed information about the recipe of the products, which is not provided by KWP data. First, some products are not only composed of legumes, like cassoulet for instance, which also contains meat and sauce. We therefore have to calculate a sub-coefficient to measure the proportion of cooked legumes used. Second, some products, such as bakery products or dehydrated meals, lose water during processing, which impacts quantities. We thus calculate a sub-coefficient to adjust quantities for those products. Third, to make the quantities comparable, we construct a conversion factor to express the quantities purchased in dry-legume equivalent quantities. Finally, based on the first three sub-coefficients, we derive an overall conversion coefficient.

• Proportion of legumes used (“*Legume_Share*”)

To determine the percentage of legumes used in each product identified, we follow a precise method. The first step is to search the product according to its name (recovered from several variables) in the INCA2 recipe database [6]. This database, produced in 2008 by Anses, the French Agency for Food, Environmental and Occupational Health & Safety, as part of the Second Individual and National Study on Food Consumption (INCA2^5^), provides 626 generic homemade recipes broken down into ingredients (and their quantification). If the product is found in the INCA2 recipe database, we record the percentage of legumes of the generic recipe and indicate the form of the legume contained in the final product (cooked, pre-cooked, dried, etc.). If the recipe is not found, we rely on other data sources. First, we look for the leading brand^6^ on the market for the product. We then search for the occurrence of leading brand product on the Open Food Facts database^7^ [7]. If the product is not found, we search on Oqali database^8^ [8]. As a last resort, we search the necessary information on the websites of manufacturers or retailers.

The percentage of legumes used can either be found directly in the databases or on the packaging. If the percentage is not specified, we infer it from the labelling information available, such as the rank of the legume in the list of ingredients and the protein content of the product. Indeed, in the ingredients statement, ingredients are listed in descending order of weight. Moreover, in some cases, the list of ingredients precisely indicates the quantity of one or several ingredients. Thus, it is possible to infer the percentage of legume ensuring that it is consistent with its rank in the ingredient list, the quantities of the other ingredients (when known) and the protein content of the final product. For example, a meal with a protein content of 3g/100g and containing chickpeas can hold a maximum of 36% of cooked chickpeas -as the protein content of cooked chickpeas is 8.3 g/100g-, and even less if other protein-carrying ingredients are used.

**Fig. 1:**
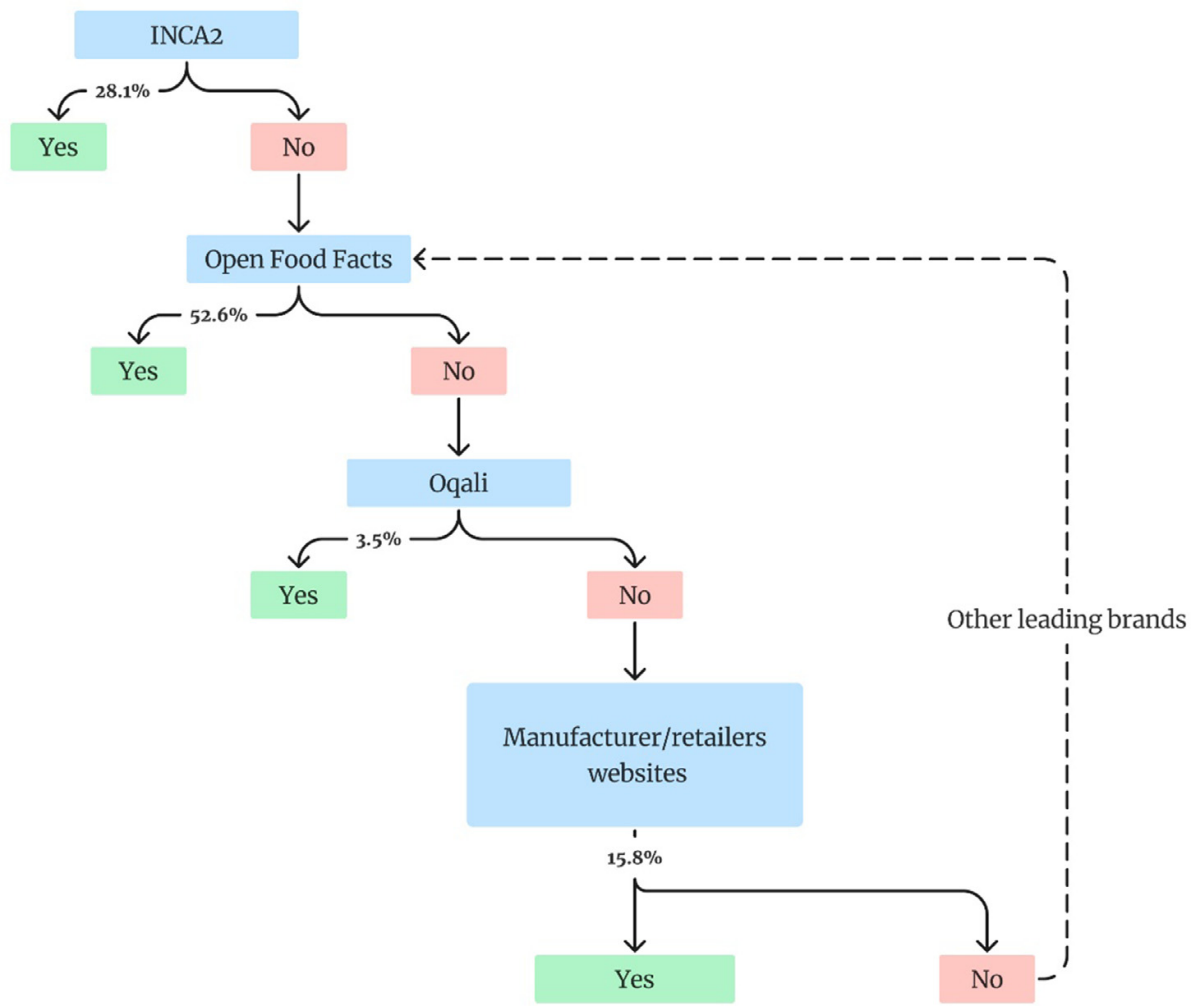
Decision process guiding the choice of information source used to determine the sub-coefficient “*Legume_Share*” for each product. [Note: Percentages are relative to the 57 products that needed information from food data to determine the sub-coefficient "Legume_Share". The sub-coefficients for the 5 remaining products are based on specific assumptions or inferred from the literature.]

If the leading brand product is not found in the databases, the second best-selling brand in terms of purchases is taken as a reference, and the same process is applied. The decision process is summarized in Fig. 1.

Due to our confidentiality agreement with KWP, we are unable to disclose the leading brands. Consequently, we cannot provide the URL links to access data relating to these products.^9^

• Rehydration coefficient (“*Rehydration_Coeff*”)

Dehydrated products such as dehydrated soup require a more specific analysis. A technical reconstitution coefficient is calculated to recover the proportion of legumes used before dehydration. To compute the coefficient, we use the information on the preparation methods that is provided on product labels. For example, 100g of dehydrated soup makes 1L of soup after reconstitution, i.e. after adding the necessary amount of water to this quantity of powder.

We also take into account the rate of return for products that lose water during manufacture (bread, biscuits). For example, to make 100g of bread, 130g of dough are necessary.

• Dry legumes conversion factor (“*Dry_Conversion_Coeff*”)

Once the percentage of legumes used in the final food product has been determined, we have to compute a coefficient to convert the quantities into dry-legume equivalent quantities. These dry legume conversion coefficients are obtained from scientific articles in nutrition or food science and grey literature (see Table 2).

• Global conversion coefficient (“*Total_Coeff*”)

**Table 2:** Inference of dried legumes conversion factor.

Type of processed legumes	Source of information	Hypothesis/Computations	Dry legumes conversion factor
Steam cooked legumes	Rio [9]		40.0%
Soaked or rehydrated or pre-steam cooked legumes	Interfel [10]		50.0%
Tofu	Rekha and Vijayalakshmi [11]	The soya juice used to make tofu is assumed to be rich, that is 14g of dry soya yield 100ml of tonyu.	71.0%
Legumes flour		Grinding has no effect on weight	100.0%
Shelled soya beans and soya flakes		Shelling and flake transformation have no effect on weight	100.0%
Soy proteins	Brasil et al. [12]	The yield of protein isolate in the industrial plant, defined as the mass of protein in the soy protein isolate per kg of soybean flour (kg/kg) divided by the mass of protein in the defatted soybean flakes per kg of soybean flour (kg/kg), is set at 73%. It is assumed that 120g of soybean yield 100g of defatted flour.	340.0%
Soy sauce	Wilson [13]		20.0%

To compute the quantity in dry-legume equivalent from the available purchased final product quantity, we then multiply the purchased quantity by the coefficient “*Total_Coeff*” defined as in Eq. (1).

(1) $$\begin{array}{c} \mathrm{Total}\_\mathrm{Coeff}=\mathrm{Legu}\mathrm{me}\_\mathrm{Share}\;\times \;\mathrm{Rehy}\mathrm{drat}\mathrm{ion}\_\mathrm{Coeff}\;\times \;\mathrm{Dry}\_\mathrm{Conv}\mathrm{ersi}\mathrm{on}\_\mathrm{Coeff} \end{array}$$

To illustrate the procedure and calculation method, an extract from the QuantiLeg dataset is provided in Table 3.

**Table 3:** Extract from QuantiLeg dataset.

Id Product	Product eng	Id pulse	Processing Type eng	Source Legume Share	Legume Share	Rehydration Coeff	Dry Conversion Coeff	Total Coeff
53	Dehydrated Moroccan soup	1	Steam cooked bean	Open Food Facts	18.0%	1000%	40%	72.0%

Product 53 refers to dehydrated Moroccan soup. This is an example of a product that undergoes water loss during processing. Since the INCA2 recipe database does not provide an average recipe for this product, we turn to an alternative source of information. First, we precisely identify the market-leading product in the KWP data^10^. Then, we search for the specific recipe in food databases available at the brand level (see Fig. 1). For this product, relevant information was found in Open Food Facts. According to the ingredient list provided, the specific branded product contains 18% cooked beans, which we use to set the “*Legume_Share*” coefficient. The information provided on the product label (available on Open Food Facts website) indicates that 100g of powder makes 4 serving sizes of 250mL, which is assumed to correspond to 1000g of liquid soup. This results in a “*Rehydration_Coeff*” of 1000%. The “*Dry_Conversion_Coeff*” is based on the literature (see Table 2), which indicates that 100g of cooked beans are obtained from 40g of dry beans. This coefficient is then set at 40%. Lastly, we calculate the “*Total_Coeff*” to be 72% from Eq. (1), meaning 72g of dry beans are required to produce 100g of the dehydrated Moroccan soup.

## Limitations

Our work faces some limitations. Firstly, in the absence of annual data on product recipes, we assume that they do not change over time. This is not an issue for classical/traditional products/recipes because their composition does not change much over time. However, recipes for other products (e.g. salads, galettes) could change over time. Hence, practitioners should use the technical coefficients of those products with caution. Secondly, a generic legume-based product in the database represents a diversity of commercialized products whose recipes may differ slightly. For example, all lentil salads are supposed to be composed with an identical proportion of lentils. The practitioner should keep in mind this approximation.

## Ethics Statement

The authors have read and followed the ethical requirements for publication in Data in Brief. They attest that the current work does not involve human subjects, animal experiments, or any data collected from social media platforms.

## Credit Author Statement

**Marine Spiteri:** conceptualization, methodology, validation, data curation, writing-original draft, writing- review and editing, supervision; **Lola Pedrini:** methodology, software, validation, data curation, writing-original draft, visualization; **Agathe Thierry:** methodology, software, validation, data curation, writing-original draft; **Valérie Orozco:** methodology, software, validation, data curation, writing-original draft, writing- review and editing; **Olivier de Mouzon:** methodology, validation, writing-original draft, writing- review and editing; **Zohra Bouamra-Mechemache:** resources, writing original draft, writing- review and editing, supervision, project administration, funding acquisition.

## Declaration of Competing Interest

The authors declare that they have no known competing financial interests or personal relationships that could have appeared to influence the work reported in this paper.

## Data Availability

Recherche Data GouvQuantiLeg (Original data).

## Footnotes

3 Stew prepared with meat (goose, duck, mutton, or pork confit) and seasoned with white beans.

4 Meat alternatives are defined as products that share attributes (such as smell, taste, texture and appearance) with meat or replace meat by providing protein in a meal context.

## Footnotes

1 BRIGHTSPACE (2022-2027) -Towards a safe and just operating space for EU agriculture- is a project that addresses the EU Horizon Europe (Topic HORIZON-CL6-2021-GOVERNANCE-01-12 - EU) led by Wageningen Economic Research. The project aims to design effective and sustainable strategies to navigate within a safe and just operating space and provides support for effective and sustainable actions (including the identification of critical pathways for technological, institutional and consumer-oriented options for EU policies in the areas of agriculture, climate change, trade, and energy). See https://brightspace-project.eu/.

2 ClasSFood is a food nomenclature, based on the French food supply and consumption, suitable to perform relevant analysis dealing with food sustainability. As a hierarchical scheme, foodstuffs are described at different levels of detail allowing for analysis of the whole diet at an aggregated level or analysis of some very specific categories described in detail.

5 INCA2 is a cross-sectional survey designed to estimate the food consumption and eating habits of individuals living in France.

6 The leading brand is defined as a food company's national brand or a retailer brand that has the largest number of purchases observed in the year 2015 in KWP data. If the product does not appear during this reference year, we use the year 2002. And if it does not appear in 2002, we use the year in which the product appeared in KWP database.

7 « Open Food Facts » is a database of food products that lists ingredients, allergens, nutritional composition and other information from food labels.

8 « Oqali » has been set up in 2008 by INRAE, Anses and the Ministries responsible for food, health and consumer affairs. Its aims at monitoring changes in processed foods supply available on the French market, by measuring nutritional quality evolution, over time (nutritional composition and labelling information).

9 However, an anonymized screen copy of the information required to calculate the sub-coefficients is available upon request.

10 As a reminder, in KWP data, a product is described by several variables, including its brand name. To respect our contractual confidentiality agreement with KWP, we cannot disclose the brand names.
